# Molecular Detection and Characterization of the *mecA* and *nuc* Genes From *Staphylococcus* Species (*S. aureus, S. pseudintermedius*, and *S. schleiferi*) Isolated From Dogs Suffering Superficial Pyoderma and Their Antimicrobial Resistance Profiles

**DOI:** 10.3389/fvets.2020.00376

**Published:** 2020-07-23

**Authors:** María S. González-Domínguez, Hernán D. Carvajal, David A. Calle-Echeverri, Danny Chinchilla-Cárdenas

**Affiliations:** ^1^INCA-CES Research Group, Veterinary Teaching Hospital, Faculty of Veterinary Medicine and Zootechny, CES University, Medellín, Colombia; ^2^Instituto Colombiano de Medicina Tropical (ICMT), CES University, Medellín, Colombia; ^3^Laboratorio de Genética Animal Mascolab S. A., Medellín, Colombia

**Keywords:** bacterial phenotyping, dogs, *mecA* gene, *Staphylococcus aureus*, *Staphylococcus pseudintermedius*, *Staphylococcus schleiferi*

## Abstract

Canine superficial pyoderma (CSP) is a bacterial infection secondary to several skin diseases of the dog. *Staphylococcus pseudintermedius*, which is a commensal bacterium of the dog's skin, is the leading agent found in dogs affected by CSP, which can progress to deep pyoderma. It is also of clinical significance because *S*. *pseudintermedius* strains carry antimicrobial resistance genes, mainly the *mecA* gene. In this descriptive longitudinal study, molecular characterization of bacterial isolates from dogs affected by CSP was performed in addition to phenotyping, antimicrobial profiling, and assessment of resistance carriage status. Fifty dogs (24 females and 26 males) attending the CES University Veterinary Teaching Hospital were included in the study. CSP was confirmed according to clinical signs and cytological examination. Swabs were taken from active skin lesions for bacterial culture, and phenotyping and antimicrobial resistance profiles were assessed using API-Staph phenotyping and the Kirby–Bauer method, respectively. We also performed molecular detection and characterization of the *mecA* and *nuc* encoding gene of coagulase-positive Staphylococci. The *mecA* gene frequency was established by qPCR amplification of a 131bp gene fragment. Data were evaluated by descriptive statistics. Erythema, peeling, pruritus, and alopecia were the predominant symptoms (72, 56, and 46%, respectively). We isolated bacteria compatible with *Staphylococcus* species from all samples tested. API phenotyping showed 83.1 to 97.8% compatibility with *S. pseudintermedius*. PCR-genotyping resulted in 15, 3, and 1 isolates positive for *S. pseudintermedius, S. aureus*, and *S. schleiferi*, respectively. Isolated strains showed high susceptibility to Imipenem, Ampicillin/Sulbactam, and Rifampicin (100, 94, and 92%, respectively). The highest resistance was against Vancomycin and Trimethoprim/Sulfamethoxazole (98 and 74%, respectively). *S. pseudintermedius, S. aureus*, and *S. schleiferi* isolates were cloned and shared 96% sequence homology. Finally, we found 62% carriage status of the *mecA* gene in isolates of CSP patients, although only 36% of the isolates were methicillin-resistant. Identification of three *Staphylococcus* species causing CSP, high-level resistance against conventional antimicrobials, and carriage of the *mecA* gene highlight the importance of performing molecular characterization of bacteria causing dermatological conditions in dogs.

## Introduction

In dogs affected by several dermatological pathologies, including atopic dermatitis, endocrine-related dermatitis, and seborrheic dermatitis, superficial pyoderma is a frequent secondary complication (1) known as canine superficial pyoderma (CSP). Almost 90% of CSP symptoms are caused by gram-positive bacteria *Staphylococcus pseudintermedius* (2). Lesions of CSP are circumscribed to the stratum corneum of the skin and hair follicle. Most clinical symptoms arise from complications with ectoparasite infestation, hypersensitivity reactions, endocrinopathies, and impaired keratinization. Common lesions of CSP patients include papules, pustules, scabs, epidermal collarettes, and focal and multifocal alopecia (1), affecting the nasal, pharyngeal, and anal regions of the skin, which are frequently colonized by *S. pseudintermedius* (3). This commensal bacterium of the dog's skin is the most frequent within the *Staphylococcus intermedius* group, although it is implicated in bacterial infections of the skin (4, 5). Other bacterial species found in healthy skin of dogs are *S. aureus, S. schleiferi* (6), and *Pseudomonas aeruginosa* (7). Because *S. pseudintermedius* occasionally colonizes the human skin causing bacterial dermatitis, it has been proposed and evidenced as a zoonotic bacterium transmitted from the dog to their owner (8–12). One critical problem in clinical settings requiring confirmatory phenotyping of staphylococci causing CSP is the inability to differentiate between *S. aureus* and *S. pseudintermedius* because of the lack of differentiation tests between these two species (13), which can result in incorrect diagnoses. Diagnostic laboratories in our clinical settings are more accustomed to phenotyping *S. aureus* isolates; although *S. pseudintermedius* is the most frequent agent causing CSP, they report *S. aureus* (14). Although the Staph-API kit can diagnose 92.49% of *Staphylococci* species, the *Staphylococcus* species of several clinical samples cannot be diagnosed. For these reasons, it is necessary to perform molecular diagnosis for differentiating between *S. aureus* and *S. pseudintermedius* in samples from CSP patients. The clinical implications of the wrong diagnosis are that several *S. pseudintermedius* strains carry resistance to methicillin (methicillin-resistant *Staphylococcus pseudintermedius*) or multidrug resistance (MDR) genes (15), resulting in lack of response to antimicrobial treatments, impairment of clinical signs, and progression of CSP toward deep pyoderma (11, 12, 16).

Several authors reported the carriage status of antimicrobial resistance-encoding genes by the genus *Staphylococcus*, the most critical being the methicillin-resistance encoding *mec* gene (11, 12, 17, 18). The *mec* gene encodes an alternative penicillin-binding protein (PBP2a) with a high affinity to β-lactamic antibiotics. This gene is encoded by the *staphylococcal chromosomal cassette mec* (*SCCmec*), which is a motile genetic element composed of three components: the *mec* complex, consisting of five classes (A, B, C, D, and E); the *ccr* complex, consisting of eight allotypes (1 to 8); and the joining (J) region (19). The variable SCCmec types: I [1B], II [2A], III [3A], IV [2B], V [5C2], VI [4B], VII [5C1], and VIII, are the result of several combinations between the five classes of the mec complex and eight ccr allotypes (20, 21). The thermonuclease encoding *nuc* gene sequence allows the design of specific primers (22, 23) for the molecular identification of *S. pseudintermedius* by PCR methods.

Based on the discrepancy regarding the etiology of CSP in our clinical setting, our working hypothesis was that *S. pseudintermedius* instead of the commonly diagnosed *S. aureus* was the staphylococcal species causing CSP in our patients. The present study aimed to identify the main microorganism present in dogs with CSP, their antimicrobial resistance profile, if there was a correlation between phenotyping, API-identification, and their molecular characterization. Finally, we evaluate the carriage status for the *mecA* gene in clinical isolates from dogs with CSP.

## Materials and Methods

This study consisted of a descriptive prospective longitudinal study, including a sample of dogs with clinical symptoms typical of CSP (*n* = 50) attending the Veterinary Teaching Hospital of University CES at Envigado, Colombia, between June 2016 and March 2017. The inclusion criteria for the dogs of the study were as follows: (1) clinical findings compatible with pyoderma; (2) presence of bacterial cocci by cytological exam and cocci growth under bacteriological culture; and (3) growth of colonies compatible with *Staphylococcus* species in blood agar and McConkey agar. The exclusion criteria were as follows: (1) cytology results indicating the presence of *Bacilli*; and (2) growth of cocci different from *Staphylococcus* species. All dogs were evaluated by the same researcher who performed the clinical exam, cytological smears from the most reliable skin lesions, and swabs sampling for bacterial culture. Samples were sent to the Veterinary Hospital Clinical Laboratory for evaluation and interpretation. Results of cytological smears and bacterial culture were available within the first hour and at 48 h after sampling, respectively. Dogs received the antimicrobial treatment according to the results of the antibiogram.

### Bacterial Culture and Phenotyping

Fifty cytological samples positive for cocci were stained with Hemacolor® for confirmation of cocci. Swab samples from these 50 dogs were cultured at 37°C/18–24 h on Blood agar and McConkey agar. Diagnosis of *Staphylococcus* species was performed by the API Staph® kit (Reference # 20500, API Systems S.A., Montalieu-Vercieu, France) (24). Briefly, isolates were subjected to 20 biochemical tests added with a homogenous bacterial suspension at 0.5 McFarland turbidity and cultured at 37°C/24 h. The VP1, VP2, NIT1, NIT2, ZYM-A, and ZYM-B reagents were added, and microorganisms were identified using the analytical catalog provided by the manufacturer. The identification was performed based on the API numerical system (24). We only selected isolates with more than 80% Staphylococcal compatibility (Table 1).

**Table 1:** Phenotyping of isolated staphylococci, according to API-Staph®.

**Phenotype**	***n***
Doubtful profile	4
Unacceptable profile	2
83.1% *S. intermedius* (if veterinary)	2
88.4% *S. intermedius* (if veterinary)	25
97.8% *S. intermedius* (if veterinary)	16
96.0% *S. intermedius* (if veterinary)	1

Antimicrobial profiles of each isolated *Staphylococcus* were established by the Kirby–Bauer method on colonies cultured in a Mueller–Hinton agar (Becton Dickinson, Heidelberg, Germany), according to the CLSI protocol (25). The antimicrobials tested were selected based on the frequency of their use for the treatment of dermatological conditions in our setting (Table 2). The minimum inhibitory concentrations were as follows: (1) Cephalexin, 30 μg; (2) Oxacillin, 5 μg; (3) Amoxicillin Clavulanate 30 μg; (4) Ampicillin sulbactam, 20 μg; (5) Enrofloxacin, 5 μg; (6) Ciprofloxacin, 5 μg; (7) Doxycycline, 30 μg; (8) Rifampicin, 5 μg; (9) Trimethoprim sulfa, 25 μg; (10) Imipenem, 10 μg; (11) Vancomycin, 30 μg.

**Table 2:** Antimicrobial sensitivity test for the isolates of Staphylococcus species.

	**Number of isolates (%)**		
**Antimicrobial agent**	**Sensitive (%)**	**Intermediate (%)**	**Resistant (%)**
Imipenem	50 (100)	0 (0)	0 (0)
Ampicillin sulbactam	47 (94)	2 (49)	1 (2)
Rifampicin	46 (92)	0 (0)	4 (8)
Cephalexin	34 (68)	2 (4)	14 (28)
Oxacillin	32 (64)	0 (0)	18 (36)
Ciprofloxacin	28 (56)	0 (0)	22 (44)
Enrofloxacin	27 (54)	1 (2)	22 (44)
Amoxicillin Clavulanate	26 (52)	9 (18)	15 (30)
Doxycycline	13 (26)	7 (14)	30 (60)
Trimethoprim-sulfamethoxazole	10 (20)	3 (6)	37 (74)
Vancomycin	1 (2)	0 (0)	49 (98)

### DNA Extraction and PCR Protocol for Staphylococcal Species

A sample of each isolate compatible with *Staphylococcus* species was harvested with a sterile loop and shed in sterile DNase-free 1.5-mL tubes containing 200-μL sterile 1 × PBS. The tube was centrifuged at 20,000 × g, and the pellet was resuspended in a 100-μL TE buffer (10 mM Tris, 1 mM EDTA), with 20-μL lysozyme. Samples were incubated at 37°C/30 min, followed by 55°C/10 min, and then centrifuged at 6,000 × g. After centrifugation, the supernatant was transferred to a new DNase-free 1.5-mL tube and was stored at −20°C until further use as a template for PCR. DNA quality was evaluated by determining the concentration through spectrophotometry using the Nanodrop 2000® its purity was analyzed by the relationship between absorbance at 260 and 280 nm.

#### Molecular Characterization

Specific primers were designed for amplifying a segment of the *nuc* gene (a gene encoding the thermostable endonuclease of coagulase-positive *Staphylococcus*) by real-time PCR sybr green I. The gene sequence for each of the three *Staphylococcus species* considered in this study was aligned using the GenBank/NCBI (26) software for identification of sequence homologies shared among the *nuc* gene (27). We used the forward and reverse primers described by Sasaki et al. (28) for the *in silico* search. Sequences were aligned in Bioedit 7.0 software (Ibis Therapeutics, Carlsbad, CA, USA) to generate a consensus sequence that was used to obtain oligonucleotide sequences for different *Staphylococcus* species using the primer3plus software. The annealing temperature was set to 60°C, to obtain PCR products of 127, 99, and 115 bp for *S. aureus, S. pseudintermedius*, and *S. schleiferi* subspecies *coagulans*, respectively (Table 3).

**Table 3:** Oligonucleotide sequence and conditions used for Staphylococci amplification of the *nuc* gene of bacteria isolated from dogs with superficial pyoderma.

**Species**	**Primer**	**Sequence**	**Gene target**	**Expected size**	**References**
*Staphylococcus aureus*	Au-nucF Au-nucR	5′-CAGAAACGGTGAAACCGAAT-′3 5′-CCATAGCGGTCTTGCTTTTC-′3	*nuc*	127pb	This Study
*Staphylococcus pseudintermedius*	Pse-nucF Pse-nucR	5′- TGATGCAGCTTTTCCGTATG -′3 5′- AAAGATGGGCAAGATGAACG -′3	*nuc*	99pb	This Study
*Staphylococcus schleiferi* subspecies *coagulans*	Sch-nucF Sch-nucR	5′- TTAAAACGACGGAAGGCAGT -′3 5′- CCAATCATACGCACACGTTC -′3	*nuc*	115pb	This Study
Internal Control *Staphylococcus species*	RNA16s-R RNA16s-R	5′- GAACCGCATGGTTCGATAGT-′3 5′- CTACGTAACGTCGCCTTGGT-′3	*16S rRNA*	109pb	This Study
*Staphylococcus species*.	Stph-mA Fw Stph-mA Rw	5′-GGCCAATACAGGAACAGCAT-′3 5′- CCCAATTTTGATCCATTTGTTG -′3	*mecA*	131pb	(29, 30) This Study

*1: One cycle at 95°C for 300 s, 40 cycles at 95°C for 10 s, 60°C for 30 s, and 65 to 95°C with 0.1°C increment during 420 s*.

#### *mecA* Gene Detection

In order to evaluate the presence or absence of the *mecA* gene in our samples, a 131-bp fragment of the gene was amplified using real-time PCR. The primers were designed based on an alignment of three *Staphylococcus* sequences available in GenBank/NCBI (*S. aureus* KP336394.1, *S. epidermidis* KP336396.1, and *S. saprophyticus* KP336397.1), following the same procedure indicated for species-specific primers. In order to confirm the identity of amplified fragments, products were sequenced and edited using the BioEdit software v7.1.316.

### The *nuc* Gene qPCR SYBR Green Amplification Protocol

Amplification was achieved using the commercial kit QuantiNova® SYBR® Green PCR Kit (Qiagen®, Germantown, USA) in a total volume of 12 μL, containing 0.5 μM of each primer and 2-μL template DNA (average DNA amount was 66.6 ng/μL). PCR was run as follows: denaturation at 95°C/5min, 40 cycles at 95°C/10 s, and 60°C/30 s. Bound SYBR Green I fluorescence was measured after each amplification cycle. Finally, a Melting Curve Analysis was performed in a temperature gradient between 65 and 95°C. Melting temperature (MT) of each amplified segment was determined and compared to the predicted MT with uMelt® (melting curve predictions software—University of UTAH®).

Amplicons were visualized in a 2.5% agarose gel electrophoresis and run at 100 V for 45 min. Fragments corresponding to the expected size (Table 3) were cut and put into 1.5-mL tubes for purification using the QIAquick Gel Extraction Kit®, according to the manufacturer's instructions. Briefly, each gel fragment was added with 6 vol QG buffer and incubated at 50°C until gel dissolution. One volume of isopropanol was added, and the resulting solution was poured into the affinity column, washed, and eluted twice with a 50-μL EB buffer. The obtained DNA was stored at −20°C until further processing.

### Sequencing Protocol

Amplicons obtained after real-time PCR were diluted at 12 μM and sequenced by Macrogen, Seoul, Korea, using the forward primer. The resulting sequences were analyzed by BLAST against the *nr* (GenBank/NCBI) database. For sequence analysis and phonogram construction, sequences in FASTA format were recorded in BioEdit v7.1.316 software (Ibis Therapeutics, Carlsbad, CA, USA) and aligned using the ClustalW program. Nucleotide Blast searches against the GenBank database in NCBI confirmed that each of the sequences generated has sequence identity scores of 96% or higher to each of the three Staphylococcal species of interest.

### Statistical Analysis

Data were consigned in Microsoft Excel (Microsoft Office); quantitative variables were analyzed by descriptive statistics, and the qualitative variables were represented in absolute and relative frequencies. To explore the possible association between clinical variables and the presence of *S. pseudintermedius*, crossed tables were performed using the IBM SPSS 22 statistical package to obtain the Pearson chi-square value. Significant *P*-value was 0.05, with 95% confidence interval.

### Institutional Board Committee Certification

The Ethics Committee of CES University approved this study. All procedures were performed according to the Code of Ethics for Animal Subject Experimentation for Veterinary Medicine (Ley 576, 2000 and Ley 84, 1989, Republic of Colombia).

## Results

**Figure 1:**
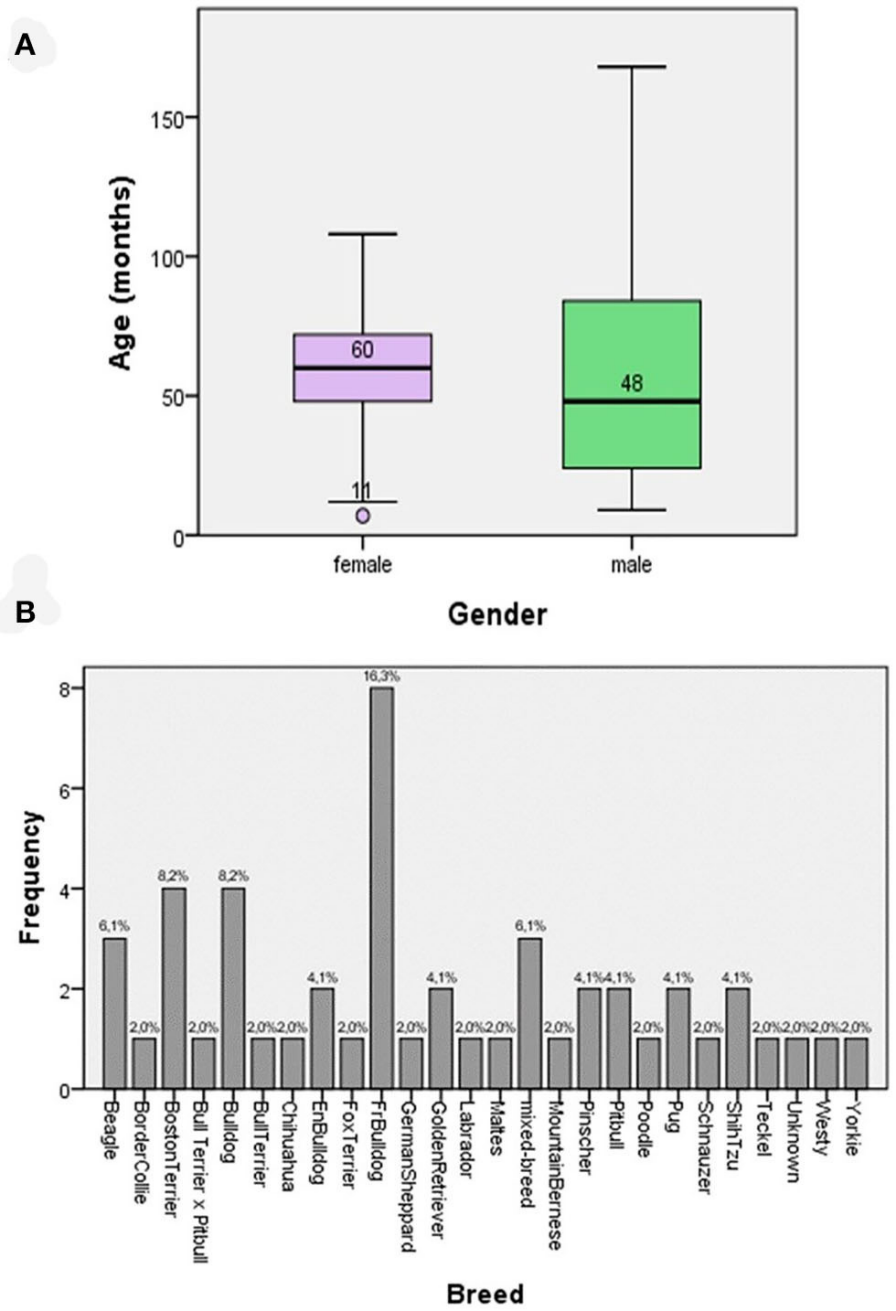
**(A)** Box and whisker plots for the age of the patients according to gender. **(B)** The frequency of breeds of patients suffering from superficial pyoderma included in the study.

Demographic data comprised 24 females and 26 males; age ranged from 7 to 168 months (average 58, and 3 and 61 months for females and males, respectively) (Figure 1A). The dog breeds are presented in Figure 1A, with most of the dogs belonging to a single breed (Figure 1B). All dogs exhibited at least one clinical sign compatible with CSP. The most frequent lesions were as follows: erythema, 36%; peeling, 28%; pruritus 23%; alopecia, 23%, epidermal collarettes, 18%; scabs, 15%; and pustules, 14% (Table 4). Thirty-three patients presented previous lesions mostly dermatological; 14 patients did not present systemic pathologies, and no data related to the presence or absence of associated skin diseases was obtained from three patients.

**Table 4:** The frequency of skin lesions in dogs suffering from superficial pyoderma.

**Skin Lesions**	***n***	**%**
Erythema	36	72
Peeling	28	56
Alopecia	23	46
Pruritus	23	46
Collarets	18	36
Scabs	15	30
Pustules	14	28
Hyperpigmentation	6	12
Hyperkeratosis	5	10
Ulcers	2	4

**Figure 2:**
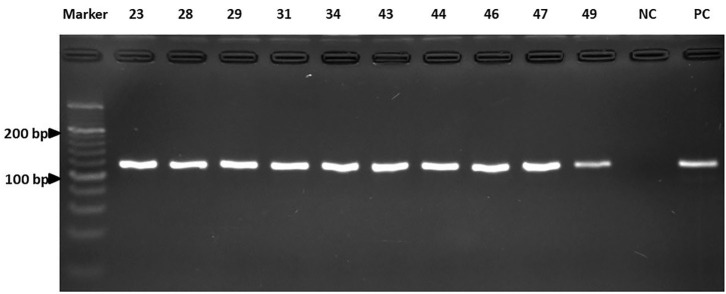
The results of the real-time PCR assay. Detection of the *nuc* gene of *S. pseudintermedius* by electrophoresis. Line A: Marker. Lines B to K: Isolates. NC, Negative control; PC, Positive control. Electrophoresis was run as indicated in Materials and Methods.

**Figure 3:**
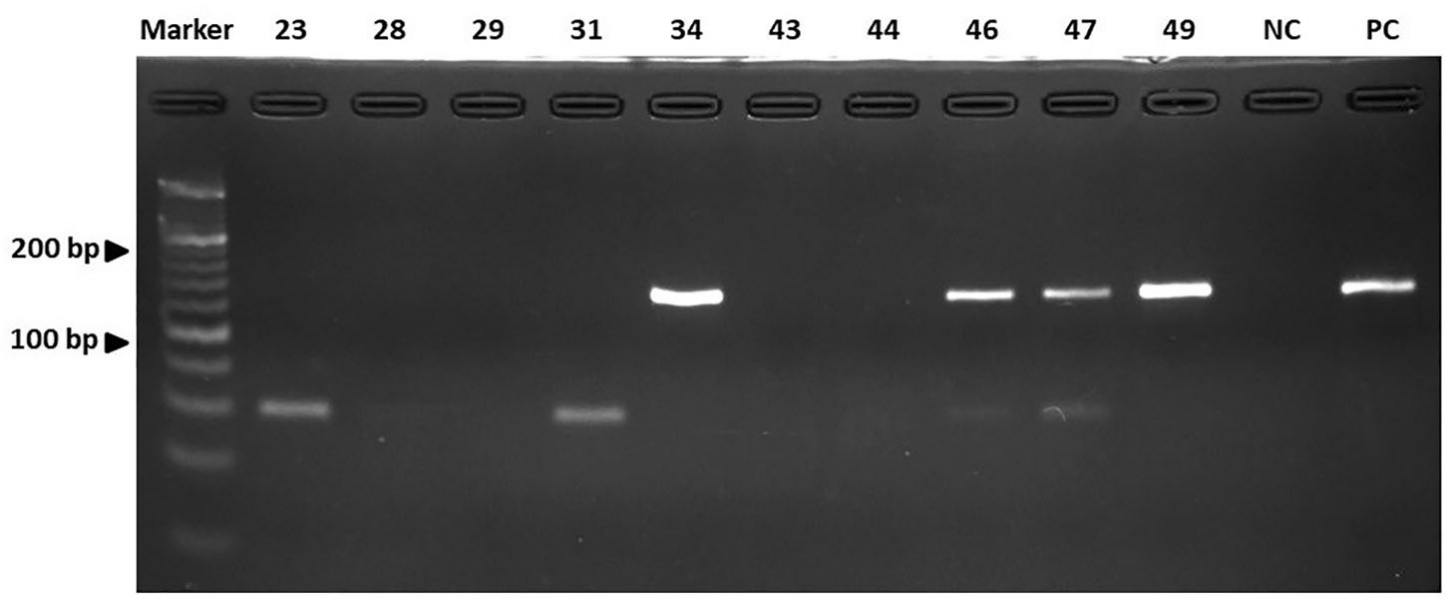
The results of the real-time PCR assay. Detection of the *nuc* gene of *S. schleiferi* by electrophoresis. Line A: Marker. Lines B to K: Isolates. NC, Negative control; PC, Positive control. Electrophoresis was run as indicated in Materials and Methods.

**Figure 4:**
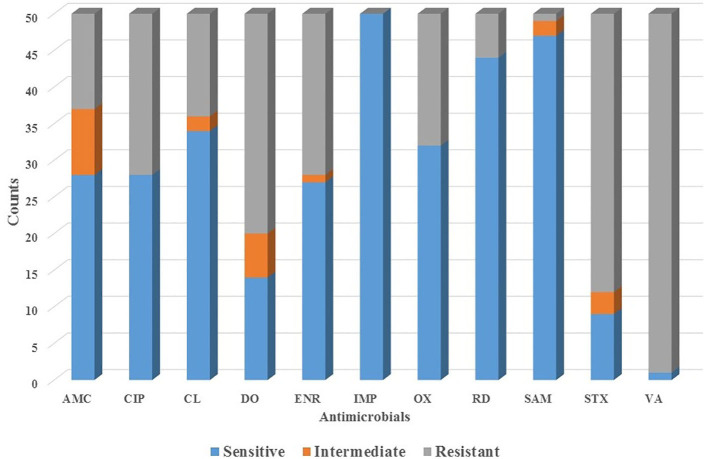
The profile of antimicrobial sensibility of Staphylococcal isolates obtained from canine patients suffering from CSP. AMC, Amoxicillin/Clavulanic acid; CIP, Ciprofloxacin; CL, Cephalexin; DO, Doxycycline; ENR, Enrofloxacin; IPM, Imipenem; OX, Oxacillin; SAM, Ampicillin/Sulbactam; VA, Vancomycin; RM, Rifampicin; TMX, Trimethoprim/Sulfonamide.

All 50 dogs tested positive for *Staphylococcus* species and were evaluated for the antimicrobial profile. Within these, 88% were positive for colonies compatible with *Staphylococcus* species within the inhibition halo, indicating the presence of at least two species of the genus *Staphylococcus* in these isolates. These samples were further processed by real-time PCR resulting in the growth of *S. pseudintermedius* (Figure 2) and *S. schleiferi* (Figure 3). The most common antimicrobial effective against the isolated strains were Imipenem, Ampicillin/Sulbactam, and Rifampicin, exhibiting 100, 94, and 92% of sensitivity, respectively (Table 3). The higher level of resistance was 98 and 74% for Vancomycin and Trimethoprim/Sulfamethoxazole, respectively (Table 3). We found 36% isolates resistant to Oxacillin (Figure 4). The *S. pseudintermedius* PCR results were significantly associated with females (*P* < 0.01) (Table 5). API phenotyping showed 83.1 to 97.8% of isolates compatible with *S. pseudintermedius*. PCR-genotyping showed 15, 3, and 1 isolates positive for *S. pseudintermedius*, the three *Staphylococcal* species, and *S. schleiferi*, respectively.

**Table 5:** Relationship between qualitative variables and the confirmed presence of *Staphylococcus pseudintermedius*.

**Variable**	**Categories**	**Presence of *S. pseudintermedius***				**Pearson chi-square**
		**No**		**Yes**		
Gender	Female	9	39.1%	14	60.9%	0.003^*^
	Male	21	80.8%	5	19.2%	
Deworming	No	4	80.0%	1	20.0%	0.325
	Yes	24	57.1%	18	42.9%	
Pathological history	No	7	53.8%	6	46.2%	0.812
	Yes	21	63.6%	12	36.4%	
Pustules	No	22	61.1%	14	38.9%	0.978
	Yes	8	61.5%	5	38.5%	
Erythema	No	10	71.4%	4	28.6%	0.354
	Yes	20	57.1%	15	42.9%	
Epidermal collarettes	No	19	59.4%	13	40.6%	0.715
	Yes	11	64.7%	6	35.3%	
Scabs	No	24	66.7%	12	33.3%	0.193
	Yes	6	46.2%	7	53.8%	
Ulcers	No	29	61.7%	18	38.3%	0.739
	Yes	1	50.0%	1	50.0%	
Scaly lesions	No	15	65.2%	8	34.8%	0.590
	Yes	15	57.7%	11	42.3%	
Alopecia	No	17	60.7%	11	39.3%	0.933
	Yes	13	61.9%	8	38.1%	
Hyperpigmentation	No	25	58.1%	18	41.9%	0.235
	Yes	5	83.3%	1	16.7%	
Pruritus	No	12	48.0%	13	52.0%	0.052
	Yes	18	75.0%	6	25.0%	
Hyperkeratosis	No	26	60.5%	17	39.5%	0.770
	Yes	4	66.7%	2	33.3%	

* *p < 0.05 statistically significant*.

**Figure 5:**
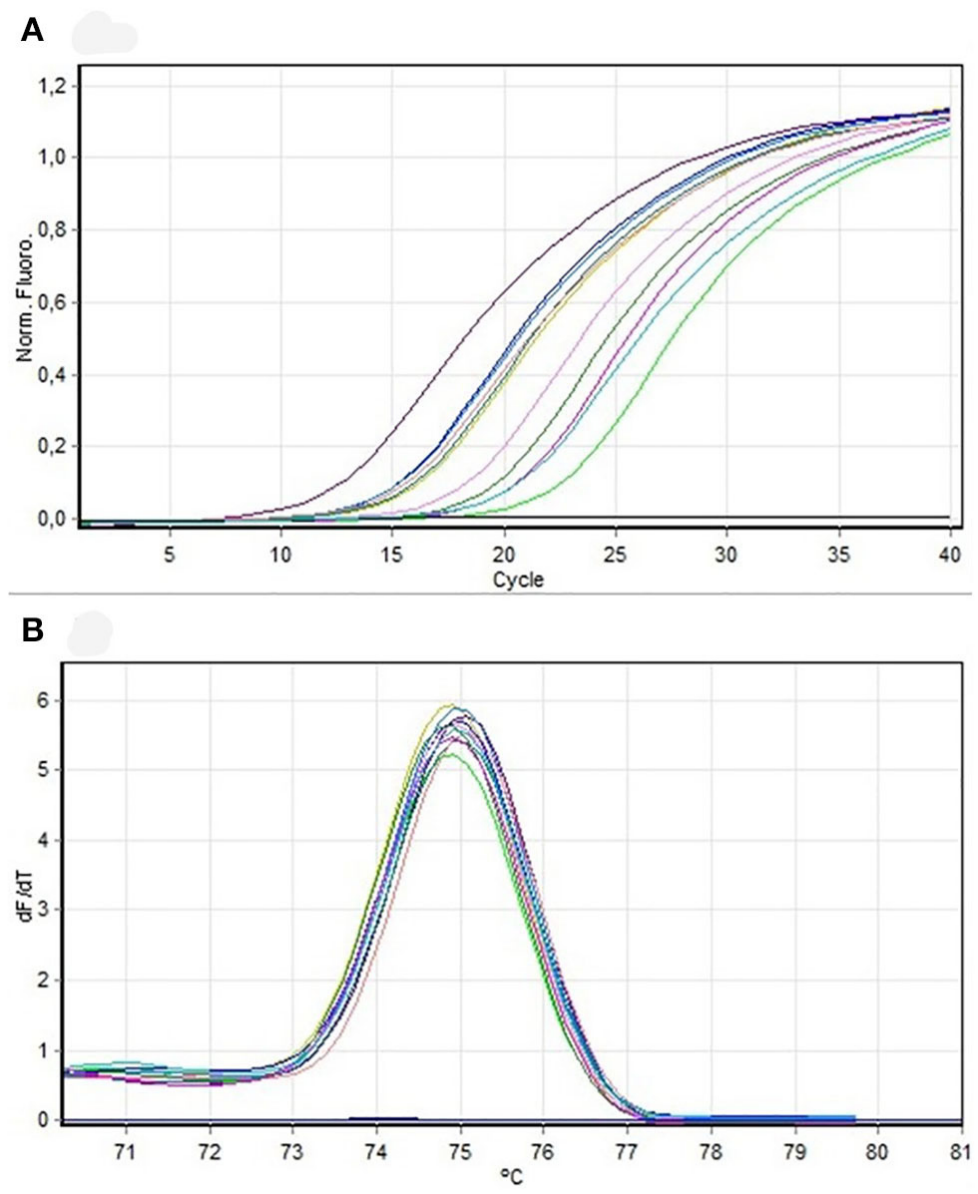
**(A)** Amplification curve. PCR mecA samples to be sequenced. **(B)** Dissociation curve. PCR mecA samples to be sequenced.

The fragment corresponding to the *mec* gene was amplified in 62% of all isolates in this study. From the total number of isolates compatible with *S. pseudintermedius* by PCR, 73.6% harbored the *mec* gene, although without statistically significant differences (*P* = 0.237, chi-squared test) (Figures 5A,B). Ten amplification fragments of the *mec* gene were sequenced, exhibiting 98.8% homology to *Staphylococcus* sequences, particularly to a region of the *mecA* gene. The sequences were aligned and compared using the ClustalW method, resulting in a complementary sequence to the *mecA* gene of the *S. aureus* strain IQH (accession number MK214488.1).

## Discussion

In this study for the first time in Latin America, the concomitant infection with two or three Staphylococcal species in dogs with CSP was reported, whereas the diagnosis of infection with one species (*S. pseudintermedius*) was reported in the remaining cases. Most previous studies reported infection with *S. aureus*, mainly. The finding of *S. pseudintermedius* in dogs with CSP provides the criteria for a more precise definition of the etiology of CSP and its corresponding treatment. *S. aureus* is the most frequent commensal found in humans, particularly in the nasal cavity of 30% healthy individuals. Within coagulase-negative staphylococci, *S. epidermidis* is most commonly isolated from nostrils, perineum, inguinal, axillary, and interdigital skin. In dogs, *S. pseudintermedius* is considered the most common coagulase-positive commensal isolated from 37 to 92% of healthy dogs, whereas *S. aureus* is only isolated in 4.3 to 12% of healthy dogs (31). Superficial pyoderma and superficial folliculitis are current entities in canine dermatology, and *S. pseudintermedius* is frequently isolated in these clinical entities. CSP is not a life-threatening condition, although it contributes to impair health and well-being because of pruritus and inflammation; it is often recurrent, resulting in repeated treatments (32). Our clinical findings are similar to the report by Khoshnegah et al. (33). The lack of association with the breed is similar to this previous report (33). Our methods do not support a possible explanation for the association between sex and pruritus.

Bacterial culture and antimicrobial sensitivity tests must be established on a routine basis for the diagnosis of CSP. In scientific literature, there is growing evidence on the resistance of *S. pseudintermedius* to methicillin [methicillin-resistant *S. pseudintermedius* (MRSP)] and other antimicrobials of beta-lactamase group, which is related to the *mecA* (methicillin-resistance) gene (32, 34, 35), which complicate the treatment of insidious infections such as those occurring in dogs suffering from deep pyoderma. In our study, 100% of dogs with CSP tested positive for *Staphylococcus* species, suggesting a high incidence of this genus in dogs with dermatologic pathologies.

The high percentage of resistance of *Staphylococcus* species isolates to Trimethoprim-Sulfamethoxazole, Vancomycin, Doxycycline, Ciprofloxacin, Enrofloxacin, and Amoxicillin/Clavulanate agrees with previous studies reporting the resistance of *Staphylococcus* species to amikacin (36), lincosamides (37), clarithromycin (38), cephalexin (39), and quinolones (40, 41). We also found a 36% resistance to Oxacillin, suggesting that these isolates probably have the *mecA* gene. Other studies had reported resistance to methicillin (17, 35, 42, 43). On the contrary, we found a high percentage of isolates exhibiting susceptibility to Imipenem, Ampicillin/Sulbactam, Rifampicin, and Cephalexin (Table 2). Other studies reported variable degrees of susceptibility of this genus to synthetic thiazole compounds (44), cationic peptides (45), naturals antimicrobial peptides and cathelicidin (46), orbifloxacin (47), marbofloxacin (48), pradofloxacin (49), and phosphomicin (50).

In most patients in our clinical setting, we have found *Staphylococcus* species isolates resistant to methicillin and other antimicrobials commonly used as therapies in dermatitis caused by *Staphylococcus* species. The persistence of resistant isolates limits the effectiveness of antimicrobials frequently used, as indicated by Cain (51). Accordingly, performing bacterial culture and susceptibility tests becomes mandatory for patients with recurrent clinical sings. Therefore, Cain (51) proposed to perform a bacterial culture and antimicrobial resistance test for all patients meeting the following criteria:

1. They are affected by recurrent pyoderma.
2. Suffering from skin infections that have not responded to appropriate empirical therapy.
3. Present clinical lesions typical of deep pyoderma, including nodules, hemorrhagic blisters, fistulas, and boils.
4. Cytological exam evidencing mixed infection by bacilli and cocci.
5. Repeated antimicrobial therapy predisposing to colonization and subsequent infection by methicillin-resistant strains.
6. Previous infection with methicillin-resistant *Staphylococci*, because colonization can persist for prolonged periods.

We propose that all those patients that meet the criteria mentioned above must undergo bacterial culture and antibiogram, which will help establish appropriate therapies, shorten diagnosis times, and shorten patients' recovery time. This procedure will be of critical importance for the outcome of the patients.

One and three of our dogs were concomitantly infected with three and two *Staphylococcal* species, *S. aureus, S. pseudintermedius*, and *S. schleiferi*, respectively. Larsen et al. (52) found that samples of dogs from several lesions resulted in the isolation of genetically different strains of *Staphylococcus* species taken from different regions of the skin, and up to four strains isolated of the same dog showed variable resistance profiles, requiring treatment with several topical, and systemic antimicrobials (52).

For achieving more accurate diagnoses of staphylococcal infections of the skin of dogs, it is essential to combine more advanced protocols such as API and PCR (17, 35). In our study, we diagnosed *S. pseudintermedius* in 44 out of 50 isolates by API, whereas PCR resulted in only 15 sample positives for *S. pseudintermedius*. However, this may be attributable to recent changes in *Staphylococcus* nomenclature that could account for erroneous identifications: most isolates identified as *S. intermedius* before 2005 now are probably *S. pseudintermedius* or *S. delphini* (1).

Molecular diagnosis identifying the 5S, 16S, and 23S ribosomal subunits, and their intergenic spaces, is one of the most useful tools in bacterial taxonomy (53, 54). PCR-based diagnosis and genome sequencing have become necessary for the study of bacterial epidemiology. Accordingly, it has been shown that *S. pseudintermedius* exhibits high genetic variability and an epidemiologic structure, suggesting the occurrence of recombination events rather than mutations. Most of the MRSP strains that arose during the last 10 years are genetically distinct regarding their antimicrobial resistance profiles, *SCCmec* content, and geographic distribution (55).

In a study performed on *Staphylococcus* isolated from dogs in New Zealand, the *mecA* gene was isolated in 38% out of 176 presumptive *S. pseudintermedius* isolates (15). On the contrary, in our study, 73.6% (19 cases) of isolates corresponding to S. *pseudintermedius* were presumptive to carry the *mec* gene. Interestingly, it has been found that *S. pseudintermedius* from companion animals was the causative agent of infection in humans (9, 11, 12, 16, 56), highlighting the importance of considering those isolates found in the present study as potential zoonotic agents. On the other hand, the *SCCmec* is a complex element. Although in the present study only the mec fragment was evaluated, its presence in our *S. pseudintermedius* isolates suggests the circulation of *Staphylococcus* strains causing infection in our population of dermatologic patients, which represents the first report in our country.

In conclusion, it is common to observe increasing cases of antimicrobial resistance of staphylococcal species to the most common antimicrobials used in the current veterinary practice, as evidenced in our study for vancomycin trimethoprim/sulfonamide and methicillin. The use of API and real-time PCR for the identification of *S. pseudintermedius, S. aureus*, and *S. schleiferi* is a valuable tool for achieving better results regarding antibiotic choice and clinical outcome. The presence of *nuc* and *mecA* genes in *Staphylococcal* isolates from dogs with dermatological pathologies warns about their potential zoonotic behavior and must be considered for preventive strategies as potential causative agents of antimicrobial resistance-gene transmission to the human population. Finally, we provide evidence that *S. pseudintermedius* was the most prevalent *Staphylococcus* species in our setting, contrary to the typical report of *S. aureus*. From the clinical point of view, to achieve a precise diagnosis of the current disease is critical for the establishment of the most appropriate therapeutic protocol. In the context of the present study, performing accurate bacterial culture and antimicrobial profiles and the molecular identification of the presence of Methicillin resistance genes in Staphylococcal isolates provide a comprehensive approach that benefits clinical diagnosis, treatment, and outcome of patients suffering CSP.

## Data Availability Statement

The raw data supporting the conclusions of this article will be made available by the authors, without undue reservation, to any qualified researcher.

## Ethics Statement

The animal study was reviewed and approved by Institutional Board on Animal Subject Experimentation of CES University (CICUA) Minute # 19 from April 26, 2016. Written informed consent was obtained from the owners for the participation of their animals in this study.

## Author Contributions

MG-D designed the study, performed patients' selection and their clinical exam for verifying inclusion and exclusion criteria, sampled the dogs, analyzed the data, wrote the manuscript, and prepared its final version. HC performed bacterial culture, cytological evaluation, and API identification, and contributed to data analysis and manuscript preparation. DC-E performed DNA extraction and molecular protocols, and contributed to data analysis and manuscript preparation. DC-C performed molecular experiments, sequencing of the genes, and data analysis and approved the final version of the manuscript. All authors contributed to the article and approved the submitted version.

## Conflict of Interest

DC-C was employed by Laboratorio de Genética Animal Mascolab S.A, Colombia. The remaining authors declare that the research was conducted in the absence of any commercial or financial relationships that could be construed as a potential conflict of interest.
